# Influence of gender on clinical presentation, management practices and outcomes of ocular syphilis

**DOI:** 10.1038/s41598-024-66412-3

**Published:** 2024-07-16

**Authors:** Melissa K. Shields, Tiago E. Arantes, Stewart R. Lake, Rubens Belfort, Cristina Muccioli, Heloisa Nascimento, Rafael de Pinho Queiroz, Daniel V. Vasconcelos-Santos, João M. Furtado, Justine R. Smith

**Affiliations:** 1 College of Medicine and Public Health, Flinders Health and Medical Research Institute, Flinders University, Flinders Drive, Bedford Park, Adelaide, SA 5042 Australia; 2 Sadalla Amin Ghanem Eye Hospital, Joinville, Santa Catarina Brazil; 3 Departmento de Oftalmologia e Ciências Visuais, Escola Paulista de Medicina, Universidade Federal de São Paulo, São Paulo, Brazil; 4 Departmento de Oftalmologia e Otorrinolaringologia, Faculdade de Medicina da, Universidade Federal de Minas Gerais, Belo Horizonte, Brazil; 5 Division of Ophthalmology, Ribeirão Preto Medical School, University of São Paulo, Ribeirão Preto, São Paulo Brazil

**Keywords:** Eye diseases, Infectious diseases

## Abstract

Ocular syphilis is a re-emerging inflammatory eye disease with a clear gender imbalance, disproportionately affecting men. We investigated the impact of gender on the presentation, management practices and clinical outcomes of this condition. Data generated from a study of patients consecutively diagnosed with ocular syphilis who attended a subspecialist uveitis service at one of four hospitals in Brazil over a 30-month period were disaggregated for analysis by gender. Two-hundred and fourteen eyes (161 men and 53 women) of 127 patients (96 men and 31 women) were included. Posterior uveitis was the most common presentation in both men and women (80.1% vs. 66.7%, *p* > 0.05), but men were significantly more likely to have vitritis as a feature of their disease (49.4% versus 28.8%, *p* = 0.019). Three eyes of women had nodular anterior scleritis (*p* = 0.015). Men were more likely to undergo a lumbar puncture to assess for neurosyphilis (71.9% vs. 51.6%, *p* = 0.048), but men and women undergoing a lumbar puncture were equally likely to have a cerebrospinal fluid abnormality (36.2% vs. 25.0%, *p* = 0.393). All patients were treated with aqueous penicillin G or ceftriaxone, and there was a trend towards more men receiving adjunctive systemic corticosteroid treatment as part of their management (65.2% vs. 46.7%, *p* = 0.071). There were no significant differences in the age of presentation, bilaterality of disease, anatomical classification of uveitis, initial or final visual acuity, and rates of ocular complications between men and women. Our findings indicate that ocular syphilis has comparable outcomes in men and women, but that there are differences in the type of ocular inflammation and management practices between the genders.

## Introduction

Syphilis is a multisystem bacterial infectious disease caused by *Treponema pallidum* that has re-emerged over the past two decades as a significant public health challenge for both men and women^1–5^. This is despite the availability of reliable prevention methods, accurate screening algorithms^6^, and effective treatment with penicillin^7^. Ocular syphilis, most commonly presenting as posterior uveitis or panuveitis, is reported in up to 4% of patients with systemic syphilis and may be the first indication of infection^8^. This once-rare disease adeptly mimics other conditions, requiring that clinicians maintain heightened suspicion in patients presenting with apparently undifferentiated uveitis.

Men are disproportionately affected by both syphilis and ocular syphilis^7,8^. In 2018, the Centres for Disease and Prevention (CDC) reported that 86% of all new cases of syphilis occurred in men^2^. A recent review of ocular syphilis showed there was a man or male preponderance in 48 of 52 case series in the literature^8^; 75.6–93.3% of patients in the three largest case series were men^9–11^. In the last five years however, there has been a surge in syphilis among women of reproductive age, accompanied by a rise in the prevalence of congenital syphilis^5,12–14^. Congenital syphilis poses a significant risk for adverse pregnancy outcomes, such as stillbirth, blindness, and lifelong disability^7^. In 2022, over 3,700 infants were born with congenital syphilis in the United States, a tenfold increase compared to 2012^13^.

Gender and sex differences have been observed in various inflammatory conditions, including some forms of infectious uveitis^15,16^. Sociocultural differences between men and women may influence pathogen exposure, such as gender-linked preferences for various domestic, occupational, and recreational activities^17,18^. Delayed health help-seeking by men has been reported in many studies globally, and this may contribute to poorer outcomes in some diseases^19^. Biological factors determined by sex include hormonal and genetic regulators of the immune system^17,18^. Sex hormones modulate the immune responses through receptors on most leukocytes, with estrogen conferring protective immunity, and progesterone and testosterone suppressing innate immune responses. Incomplete inactivation of the X-chromosome and genetic polymorphisms on the sex chromosomes also contribute to the robustness of the immune response and infection susceptibility and severity. Thus, females generally mount stronger immune responses to foreign and self antigens, while males exhibit heightened vulnerability to bacterial infections^20^.

With the changing landscape in the prevalence of syphilis having major implications for public health care, it is important to consider the differences in clinical features and disease management between men and women. However, while several large studies have assessed the clinical presentation and outcomes of ocular syphilis^10,21,22^, the potential impact of gender on this vision-threatening manifestation of the infection remains unknown. In this research, we disaggregated one of the largest datasets collected from patients with ocular syphilis^10^ and analyzed that dataset by gender, allowing us to compare the ocular involvements, management practices, and complications and visual acuity (VA) outcomes between men and women.

## Methods

Data were previously collected retrospectively from patients diagnosed with ocular syphilis at the uveitis services located at four public hospitals in Brazil (specifically, Hospital das Clínicas de Ribeirão Preto, Fundação Altino Ventura, Escola Paulista de Medicina—Hospital São Paulo, and Hospital São Geraldo/Hospital das Clínicas da UFMG) between January 2013 through July 2015 as part of a published study^10^. The study was approved by the human research ethics committee at each institution, i.e. Comitê de Etica em Pesquisa do Hospital das Clínicas de Ribeirão Preto (approval number: 42370715.2.0000.5440), Comitê de Ética em Pesquisa da Fundação Altino Ventura (approval number: 017/2012), Comitê de Etica em Pesquisa da UFMG (approval number: 97794818.4.0000.5149), and Comitê de Etica em Pesquisa da UNIFESP/Hospital São Paulo (approval number: 045038/2016). The requirement for informed consent was waived by Comitê de Etica em Pesquisa do Hospital das Clínicas de Ribeirão Preto, Comitê de Ética em Pesquisa da Fundação Altino Ventura, Comitê de Etica em Pesquisa da UFMG, and Comitê de Etica em Pesquisa da UNIFESP/Hospital São Paulo, because the clinical data were collected retrospectively and analysed without identifying information. All the study methods were performed in accordance with relevant regulations and guidelines.

In the published study^10^, standardised data collection sheets were used to collect clinical information that included age, gender, treponeme and human immunodeficiency virus (HIV) serologies, results of cerebrospinal (CSF) analysis, best-corrected Snellen VA and other clinical signs, management practices, and ocular complications. Other variables including demographic information (i.e. education, income, and vocation), past history of previous syphilis and details of HIV disease, and for women, pregnancy status, were not collected. The degree and location of the ocular inflammation were classified anatomically in accordance with the Standardization of Uveitis Nomenclature (SUN) Anatomic Classification of Uveitis^23^. Characteristic clinical presentations (e.g. acute syphilitic posterior placoid chorioretinitis) were not recorded. Ocular syphilis diagnosis required confirmation of systemic *T. pallidum* infection, as indicated by a reactive treponemal serological test in addition to: (1) a reactive non-treponemal serological test; (2) an abnormal CSF; and/or (3) resolution of clinical findings following treatment with aqueous penicillin G or ceftriaxone. Abnormal CSF was defined as reactive venereal disease research laboratory (VDRL) and/or greater than 4 leukocytes/mm^3^ and protein concentration greater than 40 mg/dl). Patients were treated with aqueous penicillin G or ceftriaxone, plus adjunctive corticosteroid therapy in selected cases.

For this report, the study data were disaggregated by gender. Continuous variables were expressed as mean ± standard deviation (SD). Categorical variables were expressed as absolute and relative frequencies. Differences for each variable between men and women were compared with the Mann–Whitney U test, Pearson’s χ^2^ test, Student’s t-test, and Fisher’s exact test. A *p* value equal to or less than 0.05 indicated a statistically significant difference. In cases of bilateral involvement, the analysis incorporated both eyes, and generalized estimating equation models were applied to address any inter-eye correlations. Statistical analyses were conducted using SPSS 20.0 for Windows (IBM SPSS Statistics, Chicago, IL).

Gender and sex are distinct terms, with sex defined by “sex chromosome complement, sex steroid hormones, and reproductive organs”^20^, and gender encompassing sociocultural factors related to the behaviours and social roles of individuals^18^. Study participants self-identified their gender, and karyotyping was not performed to determine sex. Thus, the terms “men” and “women”, not “male” and “female”, are used in this work.

## Results

Clinical data were previously collected from 127 consecutive patients diagnosed with ocular syphilis^10^. Based on self-stated gender, this group of patients included 96 (75.6%) men, 31 (24.4%) women, and no transgender persons. There were 161 eyes of men and 53 eyes of women. Bilateral disease occurred at similar rates in men (67.7%) and women (71.0%). The mean duration of symptoms at diagnosis was 2.8 months and was similar between men and women. There was no difference in the serum titres of the non-treponemal test between men and women. Men had a significantly higher likelihood of being HIV-infected compared to women, with rates of 41.8% and 12.0% respectively (*p* = 0.006). HIV testing was either omitted or results unavailable for 17 men (17.7%) and 6 women (19.3%). An overview of the baseline characteristics of men and women study participants is provided in Table 1.

**Table 1:** Demographics and baseline clinical characteristics of patients with ocular syphilis.

Characteristic	All (n = 127)	Women (n = 31)	Men (n = 96)	*p* value
Age (years) at diagnosis, Mean ± SD (median, range)	46.6 ± 13.5 (48.0, 22–88)	40.0 ± 12.5 (50.0, 24–70)	45.83 ± 13.7 (47, 22–88)	0.256*
HIV-positive, n (%) [n = 104, 25 women, 79 men]	36 (34.6)	3 (12.0)	33 (41.8)	0.006†
Bilateral involvement	87 (68.5)	22 (71.0)	65 (67.7)	0.734†
Duration (months) of symptoms at diagnosis, Mean ± SD [n = 123, 30 women, 93 men]	2.8 ± 6.3	1.8 ± 2.7	3.1 ± 7.1	0.252‡
Positive serum treponemal test, n (%)	127 (100)	31 (100)	96 (100)	NA
Titer of serum non-treponemal test, ≤ 1:128, n (%)	1:64 (1:1–1:2048) 55 (43.3)	1:64 (1:1–1:2048) 13 (41.9)	1:128 (1:1–1:1024) 42 (43.8)	0.435‡ 0.859†
CSF abnormality, n (%) [n = 85, 16 women, 69 men]	29 (34.1)	4 (25.0)	25 (36.2)	0.393†
Corticosteroid treatment [n = 122, 30 women, 92 men]	74 (60.7)	14 (46.7)	60 (65.2)	0.071†

n = 127 patients, 31 women and 96 men, unless otherwise stated

*NA* not applicable, *SD* standard deviation, *CSF* cerebrospinal fluid.

* Student’s t-test.

^†^ Pearson’s chi square test.

^‡^ Mann–Whitney U test.

Posterior uveitis was the predominant type of uveitis in both men (80.1%) and women (66.7%). Although there was no difference in the anatomical classification of uveitis, men were more likely to present with vitritis (49.4% vs. 28.8%, *p* = 0.019). This gender difference remained significant after correcting for HIV co-infection (*p* = 0.016). Other posterior segment clinical signs including retinal vasculitis, retinal necrosis, choroiditis, and papillitis were similar between men and women. Three eyes of HIV-negative women, but no eyes of men, had nodular anterior scleritis (*p* = 0.015). These patients were initially thought to have non-infectious scleritis and treated with an oral non-steroidal inflammatory drug or corticosteroid. Tests for non-infectious causes were negative, including serum anti-nuclear antibody, rheumatoid factor, and anti-neutrophil cytoplasmic antibody, plus the tuberculin skin test. Table 2 outlines the clinical manifestations of ocular syphilis by gender at presentation.

**Table 2:** Presenting ocular features in patients with ocular syphilis.

Ocular feature	All (n = 214)	Women (n = 53)	Men (n = 161)	*p* value
Anatomic classification of uveitis^23^, n (%) [n = 212 eyes: 51 eyes of women, 161 eyes of men]‡				
Anterior	13 (6.1)	4 (7.8)	9 (5.6)	0.087*
Intermediate	18 (8.5)	4 (7.8)	14 (8.7)	
Posterior	163 (76.9)	34 (66.7)	129 (80.1)	
Panuveitis	18 (8.5)	9 (17.6)	9 (5.6)	
Anterior segment findings, n (%)				
Anterior chamber cells	96 (44.9)	29 (54.7)	61 (41.2)	0.083*
Keratic precipitates	42 (19.6)	12 (22.6)	30 (18.6)	0.703*
Posterior synechiae	24 (11.2)	4 (7.5)	20 (12.4)	0.488*
Cataract	16 (7.5)	6 (11.3)	10 (6.2)	0.551*
Conjunctival hyperemia	8 (3.7)	3 (5.7)	5 (3.1)	0.806*
Keratitis	4 (1.9)	1 (1.9)	3 (1.9)	> 0.999†
Nodular scleritis	3 (1.4)	3 (5.7)	0 (0.0)	0.015†
Posterior segment findings, n (%) [n = 212 eyes: 51 eyes of women, 161 eyes of men]§				
Vitritis	94 (44.3)	15 (28.8)	79 (49.4)	0.019*
Papillitis	68 (32.1)	15 (28.8)	53 (33.1)	0.684*
Retinal vasculitis	58 (27.4)	11 (21.2)	47 (29.4)	0.547*
Retinitis	54 (25.5)	13 (25.0)	41 (25.6)	0.796*
Choroiditis/chorioretinitis	30 (14.2)	8 (15.4)	22 (13.8)	0.624*
Cystoid macular oedema	11 (5.2)	3 (5.8)	8 (5.0)	0.678*
Exudative retinal detachment	9 (4.2)	2 (3.8)	7 (4.4)	0.752*
Epiretinal membrane	6 (2.8)	0 (0.0)	6 (3.8)	0.340†
Rhegmatogenous retinal detachment	3 (1.4)	0 (0.0)	3 (1.9)	0.999†
Choroidal neovascularization	2 (0.9)	2 (3.8)	0 (0.0)	0.059†
Neuroretinitis	1 (0.5)	0 (0.0)	1 (0.6)	> 0.999†

n = 214 eyes, 53 eyes of women and 161 eyes of men, unless otherwise stated.

*Generalized estimating equation models.

^†^Fisher’s exact test.

^‡^Two eyes with isolated scleritis were excluded from the classification.

^§^Evaluation of posterior segment was not possible in 2 eyes (0.9%) of 2 patients due to media opacities.

Men were more likely to undergo a lumbar puncture (LP) to assess for the presence of neurosyphilis (71.9% vs. 51.6%, *p* = 0.048). A CSF abnormality was detected in a similar number of men and women (36.2% vs. 25%, *p* = 0.393) who underwent a LP. Vitritis was not associated with the CSF result (*p* = 0.644) or the decision to perform a LP (*p* = 0.951). There was no association between HIV infection and the decision to perform a LP (*p* = 0.349). All men and women were treated with aqueous penicillin G or ceftriaxone. Seventy-four patients were treated with adjunctive corticosteroid, given by the systemic route in 73 (98.6%). There was a non-significant trend towards men being more likely to receive corticosteroid treatment (65.2% of men and 46.7% of women, *p* = 0.071).

**Figure 1:**
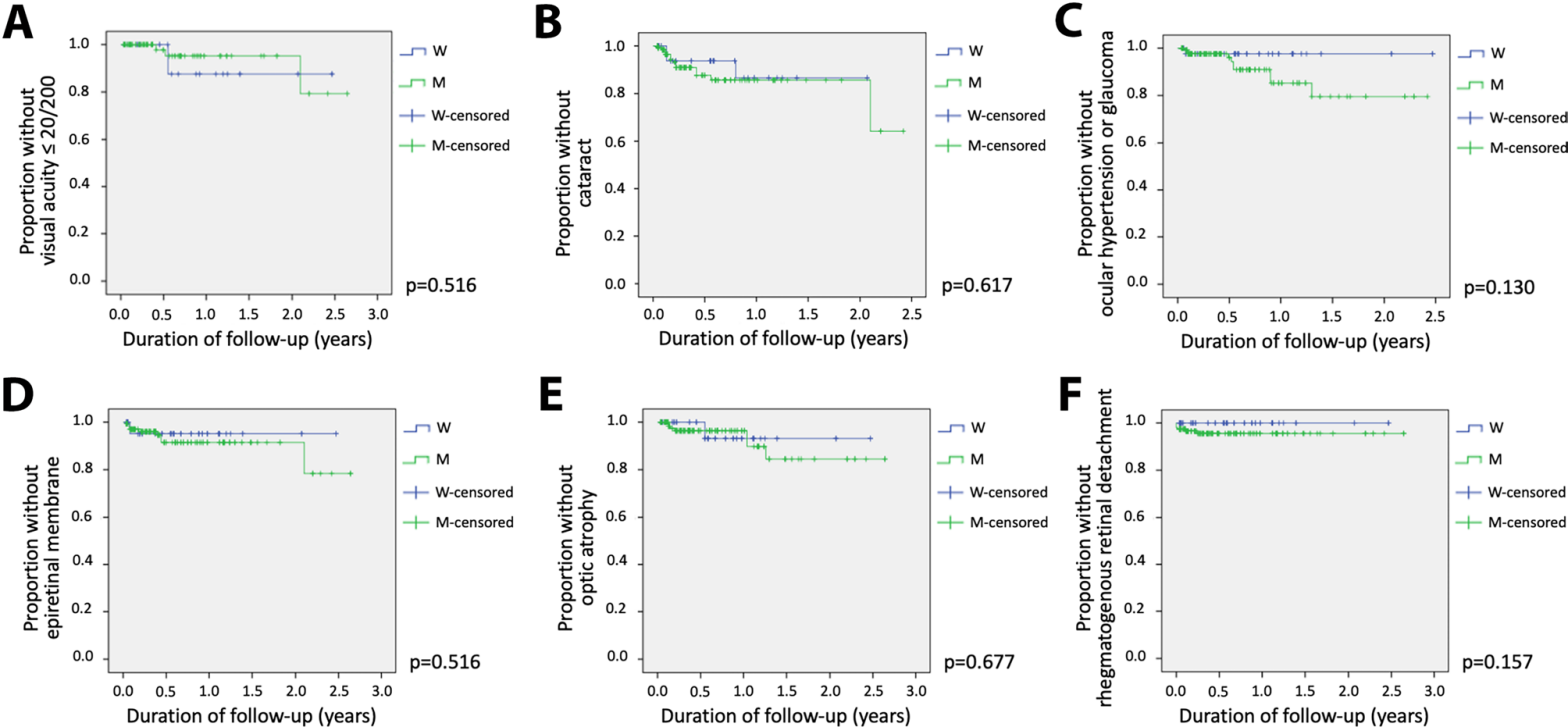
Kaplan–Meier plots showing the proportion of men versus women with ocular syphilis who remained free from the following outcomes over time, measured in years: (**A**) visual acuity ≤ 20/200; (**B**) cataract; (**C**) ocular hypertension or glaucoma; (**D**) epiretinal membrane; (**E**) optic nerve atrophy; (**F**) rhegmatogenous retinal detachment. Time 0.00 on the x-axis corresponds to the day of the first ophthalmic examination.

Of the initial 214 eyes, 50 eyes (94.3%) from women and 154 eyes (95.6%) from men were assessed in follow-up. Kaplan–Meier analysis showed that the cumulative risks of VA loss of 20/200 or more, and ocular complications including cataract, ocular hypertension or glaucoma, epiretinal membrane, optic nerve atrophy, and rhegmatogenous retinal detachment, were not significantly different between men and women (Fig. 1). Incidence rates for ocular complications and VA loss for eyes by gender are outlined in Table 3.

**Table 3:** Ocular complications and loss of best-corrected visual acuity for eyes of patients with ocular syphilis.

Complication	All (n = 204)				Women (n = 50)				Men (n = 154)			
	Number at risk	Number of events	Incidence rate (per eye-year)	Poisson Exact CI 95%	Number at risk	Number of events	Incidence rate (per eye-year)	Poisson Exact CI 95%	Number at risk	Number of events	Incidence rate (per eye-year)	Poisson Exact CI 95%
Cataract	185	16	0.18	0.10–0.28	42	3	0.16	0.03–0.37	143	13	0.2	0.11–0.34
Epiretinal membrane	200	11	0.09	.04–0.16	50	2	0.06	0.01–0.21	150	9	0.11	0.05–0.21
Glaucoma/ocular hypertension	202	11	0.10	0.05–0.17	50	1	0.03	0.001–0.16	152	9	0.11	0.05–0.22
Optic nerve atrophy	204	9	0.07	0.03–0.14	50	2	0.06	0.01–0.21	154	7	0.08	0.03–0.17
Rhegmatogenous retinal detachment	204	6	0.05	0.02–0.11	50	0	0	NA	154	6	0.07	0.03–0.15
Cystoid macular edema	191	0	0	NA	47	0	0	NA	144	0	0	NA
Choroidal neovascularization	200	0	0	NA	48	0	0	NA	152	0	0	NA
Retinal vascular occlusion^b^	202	0	0	NA	50	0	0	NA	152	0	0	NA
Visual acuity												
≤ 20/50	69	4	0.10	0.03–0.25	10	0	0	NA	59	4	0.11	0.03–0.29
≤ 20/200	126	5	0.06	0.02–0.14	33	2	0.08	0.01–0.03	93	3	0.05	0.01–0.15

n = 204 eyes, 50 eyes of women and 154 eyes of men.

*NA* not applicable.

*One patient had retinal vascular occlusion secondary to diabetic retinopathy that is not included here.

^†^Ocular hypertension was recorded when IOP exceeded 21 mmHg by Goldmann applanation tonometry. Glaucoma was diagnosed when the raised IOP was associated with characteristic glaucomatous field defects and/or retinal nerve fibre layer thinning.

Visual acuity was initially reduced to 20/50 or worse in 62.1% of men and 77.4% of women, and to 20/200 or worse in 40.4% of men and 34.0% of women. Visual acuity at 4–8 weeks was 20/50 or worse in 38.3% of men and 48.0% of women. Additionally, it was 20/200 or worse in 18% of both men and women. None of these differences were statistically significant, although there was a trend towards women being more likely than men to have reduced VA at presentation (*p* = 0.071). There was also no difference in the change in VA between men and women. Initial, 4–8 weeks and change in VA are presented in Table 4.

**Table 4:** Initial and 4–8 week best-corrected visual acuity and visual acuity change for eyes of patients with ocular syphilis.

Initial visual acuity	All (n = 214)	Women (n = 53)	Men (n = 161)	*p* value
≤ 20/50	141 (65.9)	41 (77.4)	100 (62.1)	0.071*
≤ 20/200	83 (38.8)	18 (34.0)	65 (40.4)	0.510*

Visual acuity at 4–8 weeks	All (n = 162)	Women (n = 43)	Men (n = 119)	*p* value
≤ 20/50	67 (41.4%)	24 (48.0%)	59 (38.3%)	0.324*
≤ 20/200	27 (16.7%)	9 (18.0%)	28 (18.2%)	0.988*
Change				
No change	74 (45.7%)	17 (39.5%)	57 (47.9%)	0.129*
Increase (≥ 2 Snellen lines)	80 (49.4%)	24 (55.8%)	56 (47.1%)	
Decrease (≥ 2 Snellen lines)	8 (4.9%)	2 (4.7%)	6 (5.0%)	

Initial: n = 214 eyes, 53 eyes of women and 161 eyes of men; 4–8 weeks: n = 162 eyes, 43 eyes of women and 119 eyes of men.

*Generalized estimating equation models.

## Discussion

In this study, we have identified certain gender differences in both the type of ocular inflammation and the management of ocular syphilis. By analyzing gender-disaggregated data from one of the largest published studies of this condition, we found that men presented with vitritis almost twice as often as women, and that women were more likely to present with scleritis than men. Men were approximately 50% more likely to have a LP and CSF investigation compared to women; however, rates of neurosyphilis were similar for both. Importantly, all men and women patients received treatment with appropriate antibiotics, and there were no differences in rates of complications or VA outcomes.

Over three-quarters of the study cohort identified as men. This is consistent with other published descriptions of patients with ocular syphilis, including the largest recent case series in which there was a gender bias of 75.6–93.3% toward men^9–11^. The overall incidence of syphilis is also higher in men, particularly among men who have sex with men (MSM), in whom the rate of primary and secondary syphilis is almost 250-times that in women^7^. This significant gender bias has been ascribed to high-risk behavioural practices and exposure risk rather than biological susceptibility^24^. A gender or sex imbalance becomes even more evident within the context of concurrent HIV infection, exemplified by a study that amalgamated data from 101 patients of whom 96.9% were described as being male^25^. In the dataset we used, men were almost four-fold more likely to be HIV-positive compared to women^10^. Pregnancy has been associated with an increased susceptibility to infectious diseases, with disease severity often increasing as the pregnancy advances^26^. The dataset used for our study did not include information about pregnancy in women patients, and thus we cannot comment on the impact of pregnancy on ocular syphilis.

Ocular syphilis can cause any type of ocular inflammation, although it most commonly presents as posterior uveitis or panuveitis^8^. While most men and women in our study presented with bilateral posterior uveitis, men were significantly more likely to have vitritis compared to women. Few studies compare the incidence of infectious vitritis by gender: across a cohort of patients with ocular toxoplasmosis stratified by gender there was no difference in the prevalence of vitritis between men and women (30.9% vs. 25.2%, respectively, *p* > 0.05)^16^. Notably, HIV-infected individuals with syphilis may initially present with unusually dense vitritis^27^. When controlling for HIV infection status, however, men still had more vitritis at presentation than women. Men experience a higher incidence and severity of bacterial infections^20^, including lower respiratory tract infections^28^, gastrointestinal infections^29^ and sepsis^20^. It follows that mechanisms leading to increased severity of other bacterial diseases in men may contribute to an increased cellular infiltrate in the vitreous when they develop syphilitic uveitis. Although not statistically significant, men waited longer than women in seeking medical care (approximately 3 months vs. 2 months), and this delay might have contributed to a difference in prevalence of observed vitritis between the genders.

Syphilitic scleritis is a rare manifestation of ocular syphilis^30^. All three eyes with scleritis in our study were nodular anterior and all occurred in HIV-negative women. Cunningham et al. noted that syphilitic scleritis is often nodular^31^, and Arruza et al. noted a reduced likelihood of an associated immune-mediated disease in patients exhibiting a nodular anterior subtype of scleritis ^32^. Studies on scleritis from any cause consistently show a higher prevalence among women, typically ranging 60–75%^32–34^. Scleritis is frequently associated with systemic diseases that have a significant woman preponderance and an increased prevalence during reproductive years, such as systemic lupus erythematosus, suggesting a potential role of sex steroid hormones in the pathogenesis of the condition^35^. Our study highlights the importance of testing for syphilis in any patient with scleritis.

Men and women in the cohort were managed differently following their diagnosis of ocular syphilis. Significantly more men were referred for a LP to evaluate for neurosyphilis, yet both genders were equally likely to have a CSF abnormality detected. Routine LP in all patients with ocular syphilis was reported practice by approximately 40% of uveitis-specialised ophthalmologists^36^, although the CDC recommends CSF examination only if there is clinical evidence of neurologic involvement^37^. ‘Posterior eye involvement’, encompassing vitritis, was identified as one specific situation in which uveitis specialists would arrange a LP for a patient with syphilitic uveitis^36^; however, vitritis was not associated with the CSF result or with the decision to perform a LP in our study. The bias in screening practices for neurosyphilis could indicate greater difficulty in clinically differentiating men with neurosyphilis or might reflect heightened vigilance in a population at greater risk of HIV co-infection. For an individual with ocular syphilis, being HIV-positive increases both the overall risk of neurosyphilis and the risk of asymptomatic neurosyphilis^38^.

All men and women in our study were treated with curative intravenous aqueous penicillin G or ceftriaxone. Adjunctive systemic corticosteroids are also utilised in the management of ocular syphilis, with approximately 80% of uveitis specialists reporting using them, usually after the start of antibiotics^36^, to control posterior segment inflammation including vitritis^8^. We found a trend towards men being more likely to receive treatment with corticosteroids, which may have been related to the higher rate of vitritis observed in men.

Our study has limitations, including the retrospective design and data disaggregation for analysis by gender, which may limit causal interpretation of associations. In addition, there was smaller representation of women compared to men, primarily due to the substantial global gender-related disparity in syphilis prevalence. Our data are also derived from a single country, potentially limiting the generalisability of our results. However, these data were collected from three states that encompassed both the most populous Brazilian states (São Paulo, Minas Gerais) and a less populated state (Pernambuco). Working across these regions, marked by a high incidence of infectious diseases including syphilis, enabled reporting of one of the largest cohorts of patients with ocular syphilis. With an even larger number of patients, one could examine gender differences by specific subtypes of ocular syphilis, such as acute syphilitic posterior placoid chorioretinitis. Finally, the dataset that we disaggregated for this research was collected one decade ago, and more recent data might show different trends as case numbers have continued to climb.

In summary, we confirmed gender differences in ocular syphilis including in the site of ocular inflammation and across management practices. Routine gender stratification in clinical studies represents an opportunity to gain insights into the intricate nexus between health and gender and signifies progress towards personalised medicine. In planning future studies in this field, collection of additional demographic data including education, income, and vocation, history of previous syphilis and details of HIV disease, and ocular imaging findings, along with pregnancy status of women, may expand the value of studies on the influence of gender in ocular syphilis.

## Data Availability

The dataset analysed during the current study is available from the authors upon reasonable request and with permission of the human research ethics committees that approved data collection.
